# Diet Composition Differentially Affects Insulin Pathway Compromised and Control Flies

**DOI:** 10.1155/2019/1451623

**Published:** 2019-01-22

**Authors:** Deyannira Otero-Moreno, Juan Manuel Murillo-Maldonado, Juan R. Riesgo-Escovar

**Affiliations:** Developmental Neurobiology and Neurophysiology Department, Instituto de Neurobiología, Universidad Nacional Autónoma de México, Campus UNAM Juriquilla, Boulevard Juriquilla #3001, Querétaro, QRO, c.p. 76230, Mexico

## Abstract

The insulin pathway is an anabolic pathway that controls, amongst other things, glucose homeostasis. It is an evolutionarily conserved pathway. Disruptions in insulin pathway functions can lead to diabetic states. Diabetes, a very common occurrence in modern life, afflicts a significant portion of the population of developed and developing countries worldwide. Yet, few studies have addressed the evolution of diabetic states on a long-term basis. Here, we cultured three different insulin pathway signaling compromised flies (heteroallelic mutant combinations, akin to diabetes mellitus type II) and wild type control flies, for the extent of one generation in different isocaloric diets fed* at libitum*, with or without extra methionine added. All fly stocks have a homogenized genetic background. We measured weight, total lipid, and carbohydrate content of adults at two different time points, and survival of adults reared in some of the different diets. Results show that, despite the fact that all diet regimes allow survival of at least a fraction of flies to adulthood, life histories are significantly different. Higher protein content diets promote better survival compared to higher percentage lipid and carbohydrate diets, and added methionine promotes survival in moderately reduced protein content diets. In mutants, survival is significantly reduced, and added methionine generally has an effect, albeit a more modest one. Our results highlight the value of higher percentage protein diets, and differences in effects in “healthy” versus “diabetic” states. They also show that added methionine, proposed as a “sensor” for protein content in food for flies, leads to differential effects depending on the adequacy of the diet regime.

## 1. Introduction

Depending on health status, same diets affect organismal metabolic performance, survival, and well-being differentially [1], and much needs to be done in order to get consistent results [2]. Heterogeneous experimental conditions and subjects' differences lead only to general conclusions [3]. Besides, little is known about long-term effects of diets of different composition with the same caloric value, especially in the diabetic states versus insulin signaling controls, throughout most or all of the life cycle.

General problems inherent in most studies are difficulty homogenizing genetic backgrounds, compliance with strict dietary regimes in the long term, and homogeneity of diabetic affliction [4]. Diabetes mellitus is a complex disease in humans. It is characterized by altered glucose levels in the blood, divided into two main types: diabetes mellitus type I, due to a deficiency in insulin production, and diabetes mellitus type II, due to resistance to insulin action in the body. While type I is normally an autoimmune disease that targets and kills the insulin producing cells in patients (beta cells in Langerhans islets in the pancreas), type II is polygenic and heavily influenced by the diet and environment. There are also rare monogenic cases, collectively termed MODY (Maturity Onset Diabetes in Young) which share characteristics of both types but that are generally caused by defects in pancreatic islet cell development and insulin secretion, more akin to diabetes mellitus type I, and generally caused by dominant mutations [5].

Several studies have demonstrated a growing number of genes and particular alleles that affect the proclivity to develop diabetes mellitus type II in humans [6], clouding results undertaken using human subjects with varying genetic backgrounds, making comparisons between studies difficult. The same is true of most diabetic animal models where genetic homogeneity is not strictly enforced [7].

Also, strict adherence to a particular dietary regime is all but impossible on a long term with experimental subjects like humans. Popular animal models, like rodents, are generally “made” diabetic (e.g., by streptozotocin treatment) and not “born” diabetic, so a confounding issue is the evolution of the diabetic condition to different degrees and tempos, even if the same dietary regime is enforced throughout.

The different degrees of affectation or the severity of the diabetic condition can also bias outcomes of long-term studies, even if all else is kept constant. Here we sought to overcome these common pitfalls by using homogeneous, genetically defined mutant hypomorphic conditions of the insulin signaling pathway to model diabetes type II (insulin resistance) together with a control strain [8], where all stocks have the same genetically homogenized background.

Since the initial characterization of the first viable mutant in the* Drosophila *insulin pathway more than twenty years ago [9], the fruit fly has become a favored organism for insulin studies [7]. The insulin pathway in flies is evolutionarily conserved and has been extensively characterized [7, 10].

We modeled different degrees of diabetic affectation using mutations in three different genetic loci, at different levels in the insulin-signaling cascade. We cultured the flies in isocaloric diets, with varying percentages of proteins versus lipids/carbohydrates, and in each case with two variants: provided or not with extra methionine, altogether employing eight different dietary regimes. Dietary methionine levels have been shown to affect lifespan in rodents and flies with some conflicting results [11–16]. Because of this, we sought to investigate whether changes in methionine content in flies' diets would significantly alter the parameters measured in our experiments and would significantly change the diabetic state, especially lifespan, that had not been investigated before.

## 2. Materials and Methods

### 2.1. Drosophila Stocks

The following mutant alleles of the insulin receptor (*Inr*), phosphatidylinositol 3' kinase catalytic subunit (*Pi3K92E*), and S6 kinase (*S6k*) genes were used in these studies:* Inr*^E19^(a chemically induced mutation not located to the coding sequence), and *Inr*^3*T*5^;* Pi3K92E*^A^ (a deletion of coding sequence in the 3' end), and *Pi*3*K*92*E*^5*W*3^ (*Pi3K92E* is also known as* Dp110*, and we will refer to it as* Dp110* in the paper); *S*6*k*^*L*-1^ (a deletion in the 5' of the coding sequence), and* S6k*^07084^ (a P element transposon insertion in the promoter region;* S6k*^07084^ is also known as* S6k*^P1713^; we will use this name in the paper), all originally obtained from E. Hafen, ETH, Switzerland, described in FlyBase [17]. All mutant alleles were backcrossed between five and ten generations to a control* yellow*,* white *(*y*,* w*, called in this paper* yw*) stock before use, to homogenize genetic backgrounds. This* yw* stock was first isogenized itself, as above, used to isogenize the mutant stocks, and used as a wild type control stock. A version of the third chromosome balancer chromosome, TM3, also described in FlyBase [17], carrying a transgene expressing the green fluorescent protein (GFP) from the actin 5C enhancer (Bloomington stock center stock number 4534), was used to balance all the mutant alleles. This balancer chromosome was also crossed to the* yw* background, as above, to homogenize the genetic background. Sharing a genetic background allows direct comparisons between all stocks and obviates the use of heterozygous siblings that may or may not share the same genetic background.

### 2.2. Genetics

In all cases, homozygous mutants are lethal, so we used the viable heteroallelic mutant combinations:* Inr*^E19^*/ Inr*^3*T*5^,* Pi3K92E*^ A^*/ Pi*3*K*92*E*^5*W*3^, and *S*6*k*^*L*-1^*/ S6kp*^1713^. We also used control* yw* flies bred alongside the mutant stocks, which had eclosed at the same time as the heteroallelic combination mutants. As these three loci act at different levels within the insulin pathway, they are different models of type II diabetes. Common phenotypes between the different viable heteroallelic combinations show general insulin pathway requirements (these flies have all the hallmarks of insulin pathway compromised flies: They are significantly smaller, and fewer larvae reach the pupal state; for example, see supplementary table 1), whereas specific phenotypes or the strength exhibited of a common phenotype might be germane to the locus or level of hypomorphy in question.

Crosses were made between heterozygous flies carrying different alleles of the same gene. Freshly hatched first instar larvae were collected, counted, separated in groups of one hundred or one hundred fifty, according to genotype (heteroallelic mutant larvae were separated from heterozygous siblings carrying a GFP-expressing balancer chromosome), and transferred to food vials with the different diets. The larvae developed in the different diets, and the number of pupal cases was counted (supplementary table 1). Freshly eclosed adults were used for experiments.

### 2.3. Diets

We used four different isocaloric diets (about 7% variation in Kcal/ Kg between diets), with caloric values ranging from 4241.2 to 4044.9 Kcal/ Kg. Diet Kcal values and composition of dry weight are as follows: Diet A: 4182.2 Kcal/Kg, with 71.72% of carbohydrates (725 mg/ Kg of wet weight), 11.57% of proteins (117 mg/ Kg of wet weight), and 16.71% of lipids (169 mg/ Kg of wet weight); Diet B (lower protein diet): 4044.9 Kcal/Kg, with 83.80% carbohydrates (840 mg/ Kg of wet weight), 5.03% proteins (50 mg/ Kg of wet weight), and 11.17% lipids (112 mg/ Kg of wet weight); Diet C (high carbohydrate, medium protein diet, and chemically defined): 4241.2 Kcal/Kg, with 81.93% carbohydrates (1172 mg/ Kg of wet weight), 4.97% proteins (71 mg/ Kg of wet weight), and 13.09% lipids (187 mg/ Kg of wet weight); and Diet D (very low protein diet and chemically defined): with 4062.7 Kcal/Kg, 83.58% carbohydrates (840 mg/ Kg of wet weight), 4.28% proteins (43 mg/ Kg of wet weight), and 12.14% lipids (122 mg/ Kg of wet weight). Diets A and B were regular laboratory food made from unrefined sugar (7.5 g), agar (2.25 g), and yeast paste (7.5 g) dissolved in 75 ml of doubly distilled water. For B, we only reduced the amount of yeast to half the normal amount (3.75 g). Diets C and D were made from chemically defined media purchased from Carolina Biological Supply (formula 4-24, number 173200). For diet C we used 15 grs of formula flakes in 75 ml of double distilled water, and, for diet D, we used half the normal amount of food flakes (7.5 g) and added 0.375 g of agar for food consistency (Table 1). In these basic four diets, we employed versions with and without an extra 7 mM added methionine. 7 mM methionine is below the level known to be toxic [13, 15, 18]. We used added methionine to test its effect in the various diets, since methionine may foster growth in some cases [12, 14–16].

### 2.4. Survival Studies

Newly hatched first instar larvae of the different genotypes under study were selected and placed in different food regime glass vials and allowed to pupate and eclose. One-day-old virgin females of the different genotypes were placed in groups of ten per vial with the different diets. Each vial contained 5 grs of the particular diet tested. Survival was surveyed every three days, and surviving flies were transferred to fresh food vials every seven to ten days for the duration of the experiment. Vials were cultured at 25°C and 40-50% relative humidity, in a 12:12 light: dark cycle throughout the experiment.

### 2.5. Total Carbohydrate, Total Lipid, and Weight Measurements

Total carbohydrate and lipid measurements were assessed in 7-10 individual flies, following Van Handel's protocols [19], as implemented in [9]. Virgin female flies were used throughout the analysis at one and five days old, cultured on the different diets. The same flies, before being processed for total carbohydrate or lipid measurements, were weighed individually using a Cahn C-31 microbalance set in the 0.1 *μ*g – 25 mg range, as reported in [20].

### 2.6. Statistics

We used two-way ANOVA with Friedman's post-hoc tests for life expectancy analysis. For the effects of diets on weight, total carbohydrates, and lipids, and effects between one- and five-day-old flies, we used ANOVA with Sidak's post-hoc tests. All tests were done as implemented in Prisma, in GraphPad.

## 3. Results

### 3.1. Diet Regimes Affect Weight (Size) of Adult Flies

**Figure 1:**
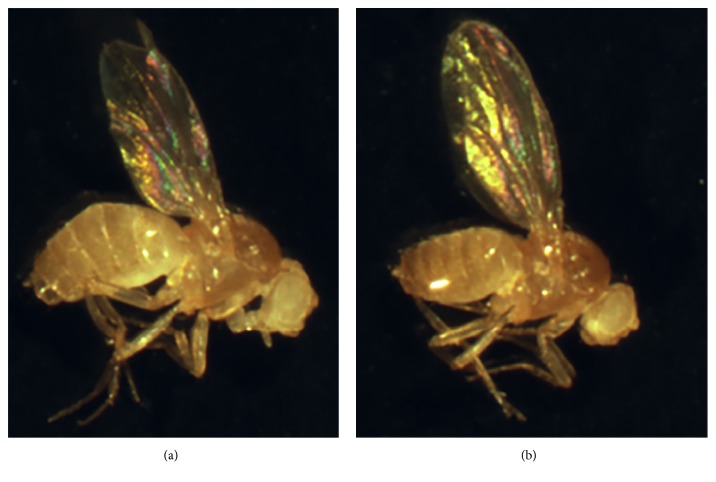
Insulin pathway compromised flies are smaller. (a) shows a one-day-old female wild type fly reared in food regime C1. Compare the size with (b), an* InR* one-day-old mutant female fly also reared in food regime C1.

For all experiments, we collected adult flies that had been reared from hatching first instar larvae to adults in the different diets. In Figure 1 we show an example of the control and heteroallelic (mutant) combinations employed in the studies (Figure 1). In order to quantify size we weighted individual flies in a microbalance at the same age. In almost all cases, mutant genotypes were significantly smaller in weight than the corresponding control (*yw*) flies. As all flies have the same genetic background, we can compare directly the results from the different genotypes.

### 3.2. Diet Regimes Differentially Affect Weight of Wild Type Adult Flies

**Figure 2:**
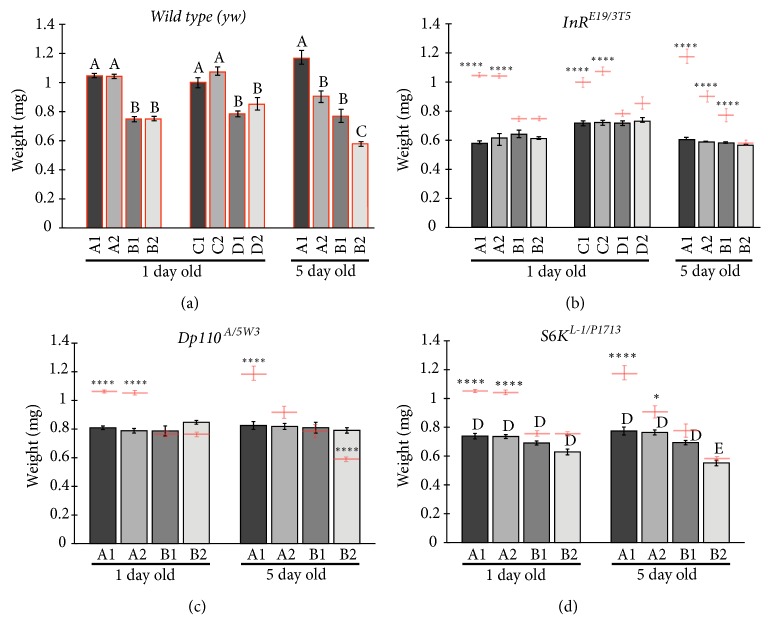
Weight measurement of individual female flies, at one and five days after emergence. In (a) we show wild type (*yw*) measurements, in (b) the* InR* heteroallelic mutant combination, in (c) the* Dp110* heteroallelic mutant combination, and in (d) the* S6K* heteroallelic mutant combination. The columns in the graph in (a) are bordered in red to show the wild type values, and the actual means plus the standard errors are copied in (b), (c), and (d) for comparison. In all graphs, the mean plus the standard error are graphed. Same letters, where present (a and d) within a panel, show not significantly different values within the columns present in the panel, but different letters evidence significantly different values; where letters above columns are absent (b and c) means that all values are not significantly different. Significant differences within comparisons between wild type and mutant values are signaled by the asterisks above the red wild type means, with *∗* p< 0.1 and *∗∗∗∗* p< 0.0001. n=12-20.

We first examined experiments conducted with the* yw* control stock (Figure 2). Reducing the amount of protein in the food (compare food A, standard laboratory food, with food where protein concentration was more than halved, foods B or D), let to significantly lower weight at birth (flies were cultured in the different foods starting as newborn first instar larvae (Figure 2(a)). This difference in weight was significant between food A and foods B and D, regardless of methionine content. In contrast, food C, whose protein content was halfway between that of food A and foods B and D, gave nonsignificant differences with food A, but significant differences with foods B and D. In other words, there are two classes of food: the higher protein content ones (foods A and C) and the lower content ones (foods B and D). This is consistent with a minimum amount of protein necessary to attain normal growth, but food C also contained over 60% more carbohydrates than food A. Carbohydrates might supplement and “rescue” a decrease in the amount of proteins. In this respect, adding extra methionine did not have a significant effect in any of the four food regimes, arguing against a direct effect of proteins without regard for carbohydrates in weight at birth.

Nonetheless, there is a caveat: wild type flies take longer to emerge from food with lower protein content (Table 2). Significantly, flies raised in foods C and D take up to 80% longer to start emerging after transfer to the food vials as newborn first instar larvae, especially in food D, the lowest protein content food. This means that the foods become increasingly less adequate for fly culturing going from food A to food D. In food C, although delayed, the food composition allows for a “normal” size, whereas that is not the case for foods B and D, regardless of the time spent before pupal emergence, arguing that overall protein content (and not only methionine enrichment) might be responsible for attaining a normal weight at birth.

Besides protein, other components (not carbohydrates or lipids, since these do not vary as much between the different food recipes) are important for faster growth: the speed of development in foods A and B is significantly faster than that of food C and D, something also seen for insulin signaling mutant flies. Not only is development slower, but the number of flies emerging is also different; after “seeding” the different food tubes with equal amounts of newborn first instar larvae, few adult flies were harvested from foods C and D, especially from food D, where, in fact, no adults ever emerged from food D for* S6K* mutants. This precluded us from studying foods C and D more extensively.

At five days after emergence, wild type flies in food without added methionine basically have the same weight, whereas those reared with added methionine show a discrete but significant reduction. Wild type flies at five days reared in the B2 food have a weight that is not different from the insulin mutant flies, suggesting that this weight (around 0.6 mg) may be close to the minimum viable weight for* Drosophila *females reared in these media, regardless of the genotype. Wild type flies reared in the A1 food have around double the weight (1.1 mg) and may represent a healthy counterpart [20].

### 3.3. Insulin Mutant Flies Do Not Distinguish between Different Food Regimes

In stark contrast to wild type flies, insulin signaling compromised flies do not have differences in weight at birth, regardless of the food regime employed: in most cases they had no significant differences between the different food regimes (Figures 2(b)-2(d)). Also, their weights at birth were, compared to wild type flies reared on identical higher protein diets, lower than control flies, similar to control flies reared on suboptimal diets. This is consistent with insulin mutant flies being unable to profit from the higher protein diets. Consistently, added methionine did not change weight at birth, either. The reduction in weight was bigger with the* InR* mutant combination, than with the other two mutants tested; this may be due to the fact that the insulin receptor is at the beginning of the insulin pathway, and the other two mutant conditions tested act downstream and conceivably affect only partially in the pathway. Alternatively, they could also represent hypomorphic conditions that are not as strong as the one seen with* InR.*

The reduction in weight at five days after emergence did not happen in the insulin mutant combinations, where the same weights are maintained at both ages. This is consistent with the idea that the mutant flies do not benefit from the differences in diets, or the added methionine, and that they are close to the minimum viable weight [20].

### 3.4. Wild Type Flies Do Not Accumulate Carbohydrates in Different Diets

**Figure 3:**
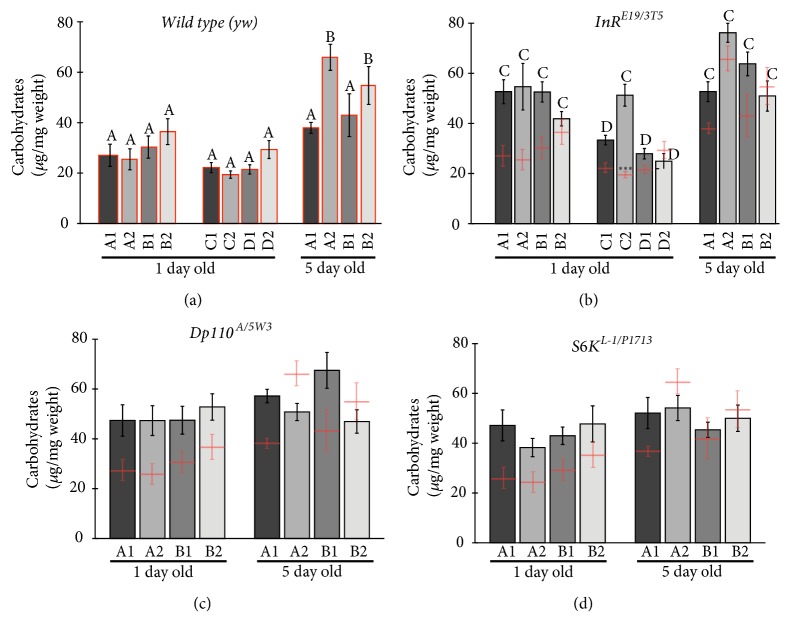
Carbohydrate measurement of individual female flies, at one and five days after emergence. In (a) we show wild type (*yw*) measurements, in (b) the* InR* heteroallelic mutant combination, in (c) the* Dp110* heteroallelic mutant combination, and in (d) the* S6K* heteroallelic mutant combination. The columns in the graph in (a) are bordered in red to show the wild type values, and the actual means plus the standard errors are copied in (b), (c), and (d) for comparison. In all graphs, the mean plus the standard error are graphed. Same letters, where present (a and b) within a panel, show not significantly different values within the columns present in the panel, but different letters evidence significantly different values; where letters above columns are absent (c and d) means that all values are not significantly different. Significant differences within comparisons between wild type and mutant values are signaled by the asterisks above the red wild type means, with *∗∗∗* p< 0.001. n=7-12.

As shown in Figure 3(a) at one day, wild type flies do not accumulate differentially carbohydrates, irrespective of the diet. At five days old, flies have accumulated significantly more carbohydrates, especially in diets with lower protein content. This is done while weight does not increase (Figure 2(a)), suggesting that flies accumulate carbohydrates at the expense of other cellular components.

### 3.5. Insulin Pathway Mutants Do Not Accumulate Carbohydrates.

At day one, most insulin pathway mutant combinations show nonsignificant but larger amounts of total carbohydrates (Figures 3(b)-3(d)). These higher levels do not augment at five days; they remain, in general, uniform. This means that, in some cases, the carbohydrates levels are similar to those in wild type flies. This last one is especially true of low protein diets.

### 3.6. Wild Type and Mutant Total Lipid Levels

**Figure 4:**
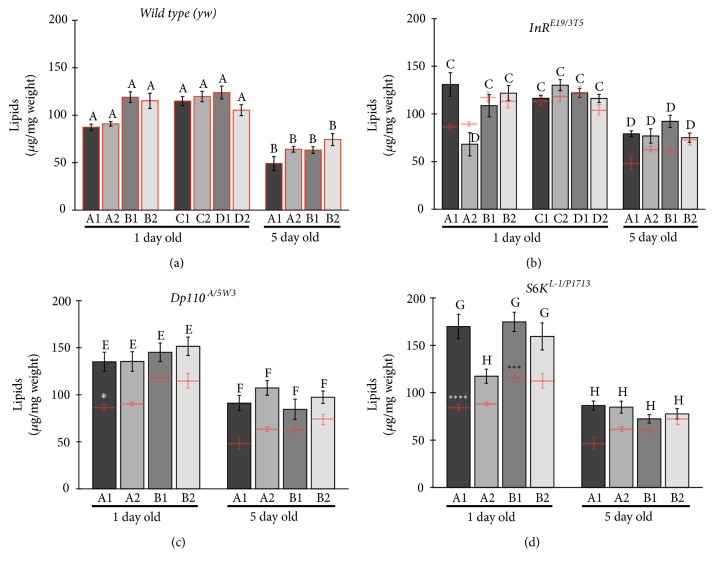
Lipid measurement of individual female flies, at one and five days after emergence. In (a) we show wild type (*yw*) measurements, in (b) the* InR* heteroallelic mutant combination, in (c) the* Dp110* heteroallelic mutant combination, and in (d) the* S6K* heteroallelic mutant combination. The columns in the graph in (a) are bordered in red to show the wild type values, and the actual means plus the standard errors are copied in (b), (c), and (d) for comparison. In all graphs, the mean plus the standard error are graphed. Same letters (a - d) within a panel show not significantly different values within the columns present in the panel, but different letters evidence significantly different values. Significant differences within comparisons between wild type and mutant values are signaled by the asterisks above the red wild type means, with *∗* p< 0.1, *∗∗∗* p< 0.001, and *∗∗∗∗* p< 0.0001. n=7-12.

In Figure 4(a), total lipid levels are roughly around 100 micrograms per milligram of wet weight in one-day-old wild type flies and diminish significantly at five days, regardless of the food regime. In contrast, insulin pathway mutant combinations accumulate higher levels of total lipids, especially in one-day-old flies. This is in line with previously published work for one-day-old insulin mutant flies and controls [9, 20]. The level of lipid in the different foods does not alter in a significant way the levels of lipids accumulated. This is consistent with lipid levels not being translated directly into accumulated lipid, something that points to a more complex metabolic regulation. Added methionine does not alter, in general, the accumulation of lipids in the different food regimes.

Lipid levels are reduced in five-day-old flies, both in mutant combinations and in control flies. Eclosing flies have, at day one, surviving larval fat cells that will lyse within a day or so and yield their contents for the young adult nutrition [21]. The fact that one-day-old flies have more accumulated lipid compared to five-day-old flies, where these larval fat cells have already suffered apoptosis and are no longer extant, most likely reflects this fact. The differences between mutant and control flies are more extreme in one-day-old flies than in five-day-old flies, and this is consistent (and likely reflects) a differential accumulation of lipids by mutant combinations, exacerbated in larval life as a consequence of the increased larval feeding behavior compared to feeding levels in adults, and the subsequent accumulation of nutrients in the larval body as opposed to the adult body, particularly lipids.

### 3.7. Life Expectancy

**Figure 5:**
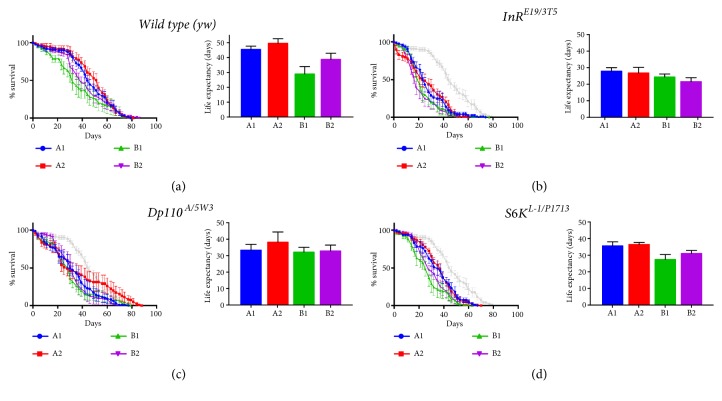
Life expectancy for flies on the different diet regimes. In (a) we show wild type (*yw*) curves (left) and life expectancy median values (right) in the different food regimes tested, in (b) the* InR* heteroallelic mutant combination curves (left) and life expectancy median values (right) in the different food regimes tested, in (c) the* Dp110* heteroallelic mutant combination curves (left) and life expectancy median values (right) in the different food regimes tested, and in (d) the* S6K* heteroallelic mutant combination curves (left) and life expectancy median values (right) in the different food regimes tested. In (b), (c), and (d) we show in grey the A1 curve for* yw* flies for comparison. In (a) the A1 food regime is significantly different from B1 (*∗∗∗*); A2 is significantly different from B1 (*∗∗∗∗*) and B2 (*∗*), and B1 and B2 (*∗∗∗*) are significantly different. In (b) A1 is significantly different from B1 (*∗*) and B2 (*∗∗∗∗*), A2 is significantly different from B2 (*∗∗*), and B1 is significantly different from B2 (*∗∗*). In (c) A2 is significantly different from B1 (*∗∗*) and B2 (*∗*). Finally, in (d) A1 is significantly different from B1 (*∗∗∗∗*) and A2 is significantly different from B1 (*∗∗∗∗*) and B2 (*∗*). Significance is as follows: *∗* p< 0.1, *∗∗* p< 0.01, *∗∗∗* p< 0.001, and *∗∗∗∗* p> 0.0001. n=10 biological replicates of 10 flies each.

How long do adult flies live on the different food regimes? We thought to investigate whether protein content had an effect, including adding extra methionine to the different food regimes. In Figure 5(a), wild type flies survived, on average, between 40 and 50 days after eclosion on a high protein diet, and this was significantly reduced when the protein content was reduced. This also shows that the high protein diets are able to sustain healthy, normal life expectancy in wild type flies. In both types of diets tested, adding extra methionine to the diet significantly increased life expectancy.

In contrast, life expectancy is reduced in the same diets tested in insulin pathway mutant combinations. This reduction is more extreme in* InR* mutants, where the difference in life expectancy is nearly half that of wild type flies. Again, this might be explained by the fact that* InR* sits at the top of the insulin-signaling cascade. In all cases, having a reduction in protein levels leads to decreased life expectancies, and, in contrast to the results in wild type flies, adding extra methionine to the food generally did not alter significantly life expectancy, although there is a nonsignificant tendency in* Dp110* mutants with a high protein diet plus added methionine (Figures 5(b)-5(d)).

Foods regimes B, with reduced protein levels compared to food regimes A, lead to significant differences in all genotypes tested, consistent with the tenet that protein levels are sensed by the different genotypes, regardless of insulin pathway functionality. This means that the sensing mechanism or mechanisms operate, as argued before, at least to a certain degree, independent of insulin signaling, as mutant stocks show differential survival.

Adding methionine to the media also leads to differences in life expectancy, especially in wild type* yw* flies, but also in some different mutant combinations, which, at face value, means that, for life expectancy, the amount of methionine in the different diets is important, and sensed as well in the hypomorphic insulin signaling backgrounds. This means that either there is at least one other mechanism independent of insulin signaling to sense methionine abundance, or else the diminished, but not null capacity to signal in the insulin mutants, is still able to signal methionine abundance, perhaps not as robustly as the wild type flies (they have fewer significant differences). As life expectancy integrates signaling throughout the life cycle of the individual, as opposed to weight, carbohydrate, and lipid measurements at a particular day, it might mean that the sum of small differences acquired throughout the life cycle leads to significant overall differences and argues that at least some degree of methionine signaling is retained in the insulin signaling compromised flies. 

## 4. Conclusions

### 4.1. Model of Diabetes

Our fly diabetes models mimic most closely diabetes mellitus type II, or resistance to insulin. Defects in the insulin-signal receiving cell are akin to insulin resistance present in type II patients, where there is no lack of insulin producing cells. Our model allows us to test long-term effects of faulty insulin signaling reception, in a controlled fashion. This allows us to study metabolic parameters and lifespan in different dietary environments and report longer-lasting trends and effects.

### 4.2. Weight

In wild type flies, diluting food leads to significant weight loss (Figure 2(a)). This might mean that flies eat a fixed amount of food regardless of nutritional content. Strikingly, this is not seen in the different mutant combinations, which never attain the same weight as controls in undiluted food (Figures 2(b)-2(d)). In fact, mutant flies' weight is similar to wild type in diluted foods, irrespective of the fact that food accessibility is* ab libitum*. In other words, mutant flies always are underweight in all food regimes tested. This might mean these reduced weights are at the lower limit, or close, to where viable flies can survive to adulthood (there is reduced survival in the diluted and “poorer” diets, see Materials and Methods).

### 4.3. Nutrients

Flies accumulate carbohydrates after eclosion in the first few days but reduce lipid levels. Lipid reduction is probably due to histolysis of the remaining larval adipocytes after eclosion, with some lipids being converted to carbohydrates. This is seen in all genotypes, and we show here that this argues for independence from insulin signaling, nutritional status, or food source (five- versus one-day-old flies). Experiments manipulating adipocyte survival should shed some light [21].

Carbohydrate accumulation might also result from adult feeding in the first few days after the pupal feeding hiatus. All our diets listed carbohydrates as major components, favoring carbohydrates consumption and carbohydrate accumulation. Experiments addressing this with drastically reduced dietary carbohydrates might prove interesting.

In general, hypomorphic insulin pathway conditions result in abnormal accumulation of carbohydrates and lipids. Here, the different diet regimes do not affect the general phenomenon. As reported earlier with flies on “normal” laboratory food [9, 20], lipids and carbohydrates are abnormally high on the first day after eclosion, with reduced weights. These hallmarks of the diabetic model are impervious to diet regimes in noncrowded conditions and, in general, at different time points. Future studies should address whether other diet manipulations can bring about significant changes.

### 4.4. Life Expectancy

Food regime A is better for rearing flies, as judged from life expectancy, in all cases. Life expectancy was significantly altered in wild type flies in all different diets (compare A1 to B1 in Figure 5(a)), with added methionine favoring longer lifespans. Regardless of abnormal carbohydrate and / or lipid accumulation, mutant flies lived significantly less (*InR* and* S6K*) and / or started dying earlier (*Dp110*), with a weaker methionine effect, if at all.

### 4.5. Methionine

Several reports show that added methionine improves reproductive capacity and life expectancy [12, 14–16], consistent with our results in wild type flies, where methionine addition increases life expectancy and carbohydrate levels in five-day-old flies. In contrast, we show no clear effect of added methionine in mutant flies, except in* Dp110 *life expectancy. Life expectancy measurement might integrate small differences, leading to overall significantly different fly life extensions, if not already metabolically compromised.

High levels of methionine in the food can be toxic [22]. Our added methionine experiments do not reach toxic levels, as flies thrive with or without added methionine, and ours are levels used in other models and studies [13, 15, 18]. Yet, in some particular cases, the added methionine may result somewhat deleterious in the mutants (for examples, see figure* Dp110* results in Figure 3 at five days, and* InR* at five days in Figures 3 and 4; in both cases with food type B).

Other papers have shown that, contrary to methionine addition to food, reduction of methionine can augment life expectancy in the context of general amino acid restriction. These authors performed experiments in flies where the insulin pathway activity was reduced by means of ectopic expression of dominant negative* InR* or* Tsc2* and found abrogated life extension by methionine restriction [13]. These conditions are different from ours, as general amino acid restriction, and even less methionine above a general reduction, might activate “survival” strategies in the organism, but that might necessitate normal insulin pathway activity.

## Figures and Tables

**Table 1:** Different diet compositions, in terms of absolute mg per 100 g of wet weight, and the percentage value of that diet component (to the right of the mg values, within parentheses).

**Food**	**Carbohydrates mg (**%**)**	**Proteins mg (**%**)**	**Lipids mg (**%**)**
**A**	72.5 (71.7)	11.7 (11.6)	16.9 (16.7)

**B**	84.0 (83.8)	5.0 (5.0)	11.2 (11.2)

**C**	117.2 (81.9)	7.1 (5.0)	18.7 (13.1)

**D**	84.0 (83.6)	4.3 (4.3)	12.2 (12.1)

**Table 2:** Days for the start of emergence of adult flies reared in the different food regimes. As can be seen in the table, with higher letter food regimes, time from newly emerged first instar larvae to the time of first emergence of adults, gets longer, for all genotypes examined.

	**Food**							
**Genotype**	**A1**	**A2**	**B1**	**B2**	**C1**	**C2**	**D1**	**D2**

***yw***	10.0	10.3	11.0	11.7	15.0	14.0	18.0	17.0

***InR*** ^***E19/3T5***^	10.3	11.2	11.5	11.5	13.5	13.5	14.0	13.0

***Dp110*** ^***A/5W3***^	10.0	10.3	11.3	11.3	12.5	14.0	15.0	15.0

***S6K*** ^***L-1/P1713***^	13.5	13.2	13.7	14.3	17.0	17.0	No emergence.	No emergence.

## Data Availability

The data used to support the findings of this study are available from the corresponding author upon request.

## Supplementary Materials

Supplementary Materials 
** Supplementary Table **1 shows the percentage of pupae from initial newly hatched first instar larvae (% pupae columns) and percentage emerged adults from initial newly hatched first instar larvae transferred to the different food regimes vials (% emerged). A1-D2 are the different food regimes vials. ND means not determined. n=2-3 independent vials per food regime for* InR*,* Dp110*, and* S6K, *n=1 for* yw* control stock.
