# Supplementary material for: Diet Composition Differentially Affects Insulin Pathway Compromised and Control Flies
Source: Biomed Res Int. 2019 Jan 22;2019:1451623. doi: 10.1155/2019/1451623 (PMC6362468; doi:10.1155/2019/1451623)
Supplement: Supplementary Materials — Supplementary Table 1 shows the percentage of pupae from initial newly hatched first instar larvae (% pupae columns) and percentage emerged adults from initial newly hatched first instar larvae transferred to the different food regimes vials (% emerged). A1-D2 are the different food regimes vials. ND means not determined. n=2-3 independent vials per food regime for InR, Dp110, and S6K, n=1 for yw control stock. [file 1451623.f1.docx]

|  | ***Inr^3T5^ / Inr^E19^*** | | ***Dp110^A^/ Dp110^5w3^*** | | ***S6K^l-1^/ S6K^P1713^*** | | ***yw*** |
| --- | --- | --- | --- | --- | --- | --- | --- |
|  | **% pupae** | **% emerged** | **% pupae** | **% emerged** | **% pupae** | **% emerged** | **% pupae** |
| **A1** | 59.6 | 40.7 | 69.4 | 53.2 | 61.5 | 43.6 | 75.0 |
| **A2** | 53.8 | 43.4 | 65.2 | 48.2 | 63.5 | 41.8 | 81.0 |
| **B1** | 57.4 | 42.0 | 66.4 | 47.5 | 55.0 | 34.8 | 49.0 |
| **B2** | 58.4 | 40.5 | 61.2 | 46.1 | 53.5 | 31.2 | 63.0 |
| **C1** | 29.7 | 17.0 | 36.0 | 25.0 | 50.0 | 26.3 | ND |
| **C2** | 32.3 | 16.5 | 30.7 | 25.0 | 59.5 | 25.5 | ND |
| **D1** | 34.7 | 23.3 | 54.3 | 23.0 | 52.0 | 3.8 | ND |
| **D2** | 32.7 | 13.0 | 55.0 | 22.0 | 56.0 | 4.5 | ND |

Supplementary Table 1.

**Supplementary Table 1.** Percentage of pupae from initial newly hatched first instar larvae (% pupae columns), and percentage emerged adults from initial newly hatched first instar larvae transferred to the different food regimes vials (% emerged). A1-D2 are the different food regimes vials. ND, not determined. n=2-3 independent vials per food regime for *InR*, *Dp110*, and *S6K,* n=1 for *yw* control stock.
